# Preliminary result of carbon-ion radiotherapy using the spot scanning method for prostate cancer

**DOI:** 10.1186/s13014-020-01575-7

**Published:** 2020-05-27

**Authors:** Yosuke Takakusagi, Hiroyuki Katoh, Kio Kano, Wataru Anno, Keisuke Tsuchida, Nobutaka Mizoguchi, Itsuko Serizawa, Daisaku Yoshida, Tadashi Kamada

**Affiliations:** Department of Radiation Oncology, Kanagawa Cancer Center, Asahi-ku, Yokohama, Kanagawa 241-8515 Japan

**Keywords:** Carbon-ion radiotherapy, Spot scanning method, Prostate cancer, Clinical outcome, Toxicity

## Abstract

**Background:**

Carbon-ion radiotherapy (CIRT) for prostate cancer was initiated at Kanagawa Cancer Center in 2015. The present study analyzed the preliminary clinical outcomes of CIRT for prostate cancer.

**Methods:**

The clinical outcomes of 253 patients with prostate cancer who were treated with CIRT delivered using the spot scanning method between December 2015 and December 2017 were retrospectively analyzed. The irradiation dose was set at 51.6 Gy (relative biological effectiveness) delivered in 12 fractions over 3 weeks. Biochemical relapse was defined using the Phoenix definition. Toxicities were assessed according to CTCAE version 4.0. *Results:* The median patient age was 70 (47–86) years. The median follow-up duration was 35.3 (4.1–52.9) months. According to the D’Amico classification system, 8, 88, and 157 patients were classified as having low, intermediate, and high risks, respectively. Androgen deprivation therapy was administered in 244 patients. The biochemical relapse-free rate in the low-, intermediate-, and high-risk groups at 3 years was 87.5, 88.0, and 97.5%, respectively (*P* = 0.036). Grade 2 acute urinary toxicity was observed in 12 (4.7%) patients. Grade 2 acute rectal toxicity was not observed. Grade 2 late urinary toxicity and grade 2 late rectal toxicity were observed in 17 (6.7%) and 3 patients (1.2%), respectively. Previous transurethral resection of the prostate was significantly associated with late grade 2 toxicity in univariate analysis. The predictive factor for late rectal toxicity was not detected.

**Conclusion:**

The present study demonstrated that CIRT using the spot scanning method for prostate cancer produces favorable outcomes.

## Background

Among cancers, prostate cancer ranks second globally in morbidity and fifth in mortality [1]. Radiotherapy (RT) and surgery have played leading roles in the radical treatment of localized prostate cancer [2]. Technological improvements in RT, such as intensity-modulated RT (IMRT) and particle therapy, can provide dose escalation without increasing toxicity in the surrounding normal tissues [3]. Several studies demonstrated that biochemical failure rates were reduced by escalating the radiation dose [4–7].

Carbon-ion RT (CIRT) for cancer treatment in humans was started in 1994 at the National Institute of Radiological Sciences (Chiba, Japan), and the first CIRT clinical trial for prostate cancer was started in 1995 [8]. CIRT offers biological and physical advantages over conventional RT with X-rays. Carbon-ion beams have an estimated threefold higher relative biological effectiveness (RBE) than X-rays [9, 10]. Regarding the physical aspect, the carbon-ion beam can create a better dose distribution based on the ability of accelerated carbon ions to release a maximum amount of energy at the end of their track, resulting in a Bragg peak [11]. These features can permit dose escalation for tumors with less toxicity in normal tissues. In fact, favorable clinical outcomes of CIRT for prostate cancer have been reported [12, 13].

The first clinical operation at the ion beam Radiation Oncology Center in Kanagawa (i-ROCK) at Kanagawa Cancer Center (KCC) was started in 2015 [14]. The i-ROCK is a compact carbon-ion facility designed by the Japanese National Institute of Radiological Sciences for widespread use and is based on a synchrotron accelerator that feeds four treatment rooms. All patients have been treated with CIRT using the spot scanning method since the opening of i-ROCK. The spot scanning method is a 3D scanning beam delivery method that uses narrow pencil beams of carbon ions to cover the entire target volume [15]. The target volume is decomposed into thin longitudinal layers that are irradiated layer by layer with the pencil beam [16]. A pencil beam can be deflected magnetically in horizontal and vertical directions to irradiate a tumor slice [17]. By reducing the energy stepwise and repeating the irradiation for each slice, a tumor can be irradiated according to its shape from the most distal end of the target to the proximal end [18–20]. This unique irradiation technique offers a more conformal dose distribution to the shape of the tumor.

In i-ROCK, the use of CIRT using the spot scanning method for prostate cancer was started in December 2015. The clinical outcomes of prostate cancer patients treated with CIRT using only the spot scanning method have not been investigated before. The present study thus aimed to analyze the efficacy and toxicities of CIRT using the spot scanning method for patients with prostate cancer.

## Methods

### Patients

In total, 253 consecutive patients with prostate cancer treated with CIRT at KCC between December 2015 and December 2017 were analyzed in the present study. Clinical records were collected in April 2020. The eligibility criteria for this study were as follows: (i) histological diagnosis of prostate adenocarcinoma, (ii) cT1bN0M0 to T3bN0M0 according to the 7th UICC classification, (iii) performance status of 0–2, (iv) age of 20 years or older, and (v) no previous treatment for prostate cancer excluding androgen deprivation therapy (ADT). The patients were classified using the D’Amico risk group classification [21]. The study was approved by the institutional review board of KCC (approval number: 2019–145). Written informed consent was obtained from all patients.

### CIRT

Patients were placed in the supine position. The patients were positioned on a vacuum mattress (BlueBAG: Elekta AB, Stockholm, Sweden) and immobilized using thermoplastic shells (Shellfitter: Kuraray, Tokyo, Japan). Enema was used before computed tomography (CT) for CIRT planning. The rectum was emptied as much as possible using a laxative and antiflatulent before each session, and enema was performed if the patient did not defecate within 24 h of treatment. The patients urinated and watered 60 min before CT. A set of CT images with 2 mm-thick slices was taken for treatment planning.

Contouring of target volumes and normal tissues was performed using MIM maestro software version 5.6. (MIM Software Inc., Cleveland, OH, USA). Dose calculation and optimization were performed using the Monaco version 5.20 system (Elekta AB).

The gross tumor volume was not defined. The clinical target volume (CTV) included the entire prostate and proximal seminal vesicles. In the case of T3b prostate cancer, the ipsilateral seminal vesicles were included in the CTV [22]. Prophylactic pelvic lymph node area was not included in the CTV [23]. Planning target volume (PTV) 1 was created by adding anterior and lateral margins of 10 mm and a posterior margin of 5 mm to the CTV. Boost therapy was performed using PTV2, in which the posterior edge was set in front of the anterior wall of the rectum to reduce the rectal dose in the ninth course of the treatment [24, 25]. The rectum was delineated as the organ at risk from 10 mm above the upper margin of the PTV to 10 mm below the lower margin of the PTV.

The total dose was set at 51.6 Gy (RBE). After the first eight fractions were delivered using PTV1, boost therapy was performed using PTV2. The PTV was covered by ≥95% of the prescribed dose, and the PTV max dose was limited to < 105% of the prescribed dose. The dose constraint for rectum was aimed at V80% < 10 ml.

CIRT was administered once daily for 4 days a week over 3 weeks. All patients were treated using the spot scanning method. CIRT was performed from both the right and left sides of the patient. One port was used for each treatment session. In each treatment session, a computer-aided online 2-D positioning system was employed to verify the positioning accuracy to less than 1 mm. In-room CT was conducted at the end of the first treatment session to confirm position accuracy. If position accuracy was confirmed, in-room CT was conducted at the fifth and ninth treatment sessions to reconfirm the patient’s position. If position accuracy was not sufficient, additional in-room CT was considered as necessary.

### Follow-up

A urologist and a radiation oncologist conducted patient follow-up at 3 month intervals for the first 3 years after CIRT and at intervals of 6 months thereafter. Prostate specific antigen (PSA) was measured at each follow-up visit. Biochemical relapse was defined using the Phoenix definition, that is, the nadir PSA level plus 2 ng/ml [26]. The duration of biochemical relapse-free survival (BFS) was calculated from the start of CIRT to the date of the event.

Toxicities were assessed according to the Common Terminology Criteria for Adverse Events version 4.0. Acute toxicity was defined as events occurring up to 3 months after the initiation of CIRT, and late toxicity was defined as events occurring after 3 months. The worst toxicity grade was considered the final grade of toxicity.

### ADT

Urologists administered ADT. ADT was not administered to low-risk patients. Neoadjuvant ADT was administered for 4–8 months to intermediate-risk patients, whereas high-risk patients received a total of 24 months of neoadjuvant plus adjuvant ADT.

### Statistical analysis

Statistical analysis was performed using STATA software (version 13.1, Texas, USA). A *p* value of < 0.05 was considered significant. BFS, and the cumulative rates of late toxicity were estimated using the Kaplan–Meier method. BFS rates in each risk group were compared via log-rank analysis. Patient characteristics were compared using Fisher’s exact test. The correlation of clinical variables with toxicities was assessed via logistic regression analysis.

## Results

### Patient characteristics

Patient characteristics are summarized in Table 1. The median age was 70 (range, 47–86) years. The median follow-up duration was 35.3 (range, 4.1–52.9) months. Among 253 patients, 8, 88, and 157 patients were classified as having low, intermediate, and high risks, respectively. The median ages in the low-, intermediate-, and high-risk group were 68 (range, 59–75), 68 (range, 48–81), and 70 (range, 47–86) years, respectively. Significant difference was observed between the intermediate- and high-risk groups (*p* = 0.034). All patients completed CIRT on schedule. ADT was administered to 244 patients, and the median duration of ADT was 22.8 (range, 2.3–116.9) months. A total of 4 patients underwent transurethral resection of the prostate (TURP) in a median of 14 (range, 9–15) years before CIRT. Of these 4 patients, 2 were classified as intermediate-risk group and 2 as high-risk group.

**Table 1:** Patient characteristics (*n* = 253)

Characteristics	n (%)
Follow-up duration, months, median (range)	24.3 (4.1–39.5)
Age, years, median (range)	70 (47–86)
T stage	
1c	49 (19.4%)
2a	79 (31.2%)
2b	35 (13.8%)
2c	53 (20.9%)
3a	27 (10.7%)
3b	10 (4.0%)
Pretreatment PSA, ng/ml, median (range)	8.6 (3.33–187)
< 10	147 (58.1%)
10 ≤ 20	73 (28.9%)
20 ≤	33 (13.0%)
Gleason score	
6	14 (5.5%)
7	117 (46.2%)
8	79 (31.2%)
9	43 (17.0%)
10	0 (0.0%)
D’Amico classification	
low	8 (3.2%)
intermediate	88 (34.8%)
high	157 (62.1%)
ADT	
none	9 (3.6%)
neoadjuvant	87 (34.4%)
neoadjuvant and adjuvant	157 (62.1%)
Complications, histories	
Diabetes mellitus	25 (9.9%)
Internal use of anticoagulants	41 (16.2%)
Benign prostate hyperplasia	18 (7.1%)
TURP	4 (1.6%)

*PSA* prostate specific antigen, *ADT* androgen deprivation therapy, *TURP* transurethral resection of the prostate

### BFS rates

Four patients died during the observation period. All patients died of other diseases, such as gastric cancer, lung cancer, hepatocellular cancer and pancreatic cancer. Prostate cancer was diagnosed as the primary type of cancer in these four patients.

**Fig. 1:**
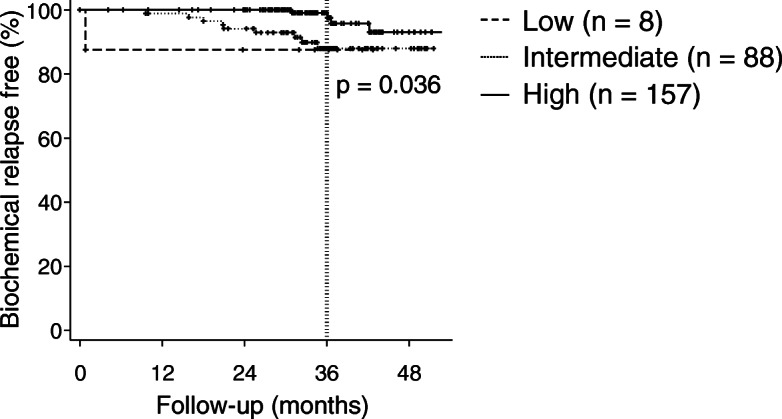
Biochemical relapse-free rate. The biochemical relapse-free rate in the low-, intermediate-, and high-risk groups at 3 years was 87.5, 88.0, and 97.5%, respectively (P = 0. 036)

The BFS rate is presented in Fig. 1**.** The BFS rates in the low-, intermediate-, and high-risk groups at 3 years were 87.5, 88.0, and 97.5%, respectively (*p* = 0.036). Biochemical relapse was observed in 14 patients. Biochemical relapse was observed at a median of 25.7 (range, 0.8–42.2) months after CIRT. Significant difference was observed between age and biochemical relapse. The median ages with and without biochemical relapse were 64 (range, 50–86) and 70 (range, 47–85) years, respectively (*p* = 0.007). Significant differences were not observed in other patient characteristics. In eleven of fourteen patients, the PSA level was decreased without any treatment such as ADT. Two patients received ADT immediately after the diagnosis of PSA failure without radiological confirmation of clinical recurrence. Clinical recurrence was observed in one patient. A patient who was classified as high-risk group experienced pelvic node and lung metastases at 36.6 months after CIRT.

### Toxicities

Table 2 shows the maximal acute and late toxicities in the present study. The acute genitourinary (GU) toxicity grades were one in 52 patients (20.6%) and two in 12 patients (4.7%). Grade 3 or greater acute GU toxicity was not observed. Among acute GU toxicities, urinary frequency was the major symptom observed. Grades 1 and 2 urinary frequency were observed in 36 (14.8%) and 12 (4.7%) patients, respectively. The second highest observed symptom was urinary stricture, with grades 1 and 2 urinary stricture in 12 (4.7%) and 3 (1.2%) patients, respectively. The acute gastrointestinal (GI) toxicity grade was one in two patients (0.8%). Both of these two patients experienced diarrhea. Grade 2 or greater acute GI toxicity was not observed.

**Table 2:** Toxicities

Toxicities				*n* = 253
Acute toxicities	Grade 0	Grade 1	Grade 2	Grade 3 or more
GU total	189 (74.7%)	52 (20.6%)	12 (4.7%)	0
urinary frequency	210 (83.0%)	34 (13.4%)	9 (3.6%)	0
urinary stricture	238 (94.1%)	12 (4.7%)	3 (1.2%)	0
urinary tract pain	249 (98.4%)	4 (1.6%)	0	0
urinary urgency	251 (99.2%)	2 (0.8%)	0	0
GI total	251 (99.2%)	2 (0.8%)	0	0
diarrhea	251 (99.2%)	2 (0.8%)	0	0
Late toxicities	Grade 0	Grade 1	Grade 2	Grade 3 or more
GU total	185 (73.1%)	52 (20.6%)	16 (6.3%)	0
urinary frequency	214 (84.6%)	26 (10.3%)	11 (4.3%)	0
hematuria	238 (94.1%)	14 (5.5%)	1 (0.4%)	0
urinary stricture	245 (96.8%)	5 (2.0%)	3 (1.2%)	0
urinary incontinence	246 (97.2%)	6 (2.4%)	1 (0.4%)	0
urinary urgency	251 (99.2%)	2 (0.8%)	0	0
GI total	238 (94.1%)	12 (4.7%)	3 (1.2%)	0
rectal hemorrhage	238 (94.1%)	12 (4.7%)	3 (1.2%)	0

*GU* genitourinary, *GI* gastrointestinal

The late GU toxicity grades were one in 52 patients (20.6%) and two in 17 patients (6.7%). Grade 3 or greater late GU toxicity was not observed. Among the late GU toxicities, grades 1 and 2 hematuria were observed in 14 (5.5%) and one patient (0.4%), respectively. Grades 1 and 2 urinary frequency were observed in 28 (11.1%) and 11 (4.3%) patients, respectively, and grades 1 and 2 urinary stricture were observed in 5 (2.0%) and 3 (1.2%) patients, respectively.

**Fig. 2:**
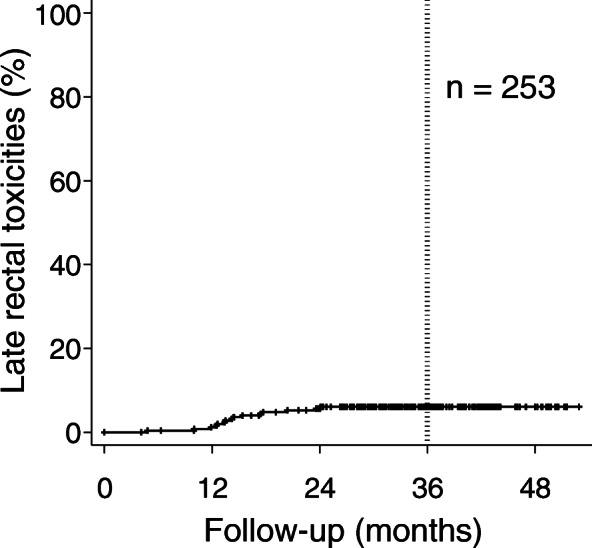
Cumulative incidence rate of late rectal toxicities. The 3-year cumulative incidence rate of any-grade late rectal toxicity was 6.1%

The late GI toxicity grades were one in 12 patients (4.7%) and two in three patients (1.2%). Grade 3 or greater late GI toxicity was not observed. All late GI toxicities were rectal hemorrhage. Grades 1 and 2 GI toxicities were observed in median durations of 14.4 (range, 10.0–23.6) and 9.1 (range, 4.8–13.4) months after CIRT, respectively (*p* = 0.167). The cumulative incidence rate of any-grade late rectal toxicity is shown in Fig. 2. The 3-year cumulative incidence rate of any-grade late rectal toxicity was 6.1%.

The predictive significance of clinical variables for the occurrence of late grade 2 toxicities was assessed via logistic regression analysis (Table 3). A history of TURP was significantly associated with the occurrence of grade 2 late GU toxicity in univariate analysis (*p* = 0.008). The number of patients who developed grade 2 GU toxicity with and without a history of TURP was 2 (50.0%) and 2 (6.0%) patients, respectively. Although diabetes mellitus (DM) was tended to be associated with the occurrence of grade 2 late GU toxicity, significant relation was not observed in univariate analysis (*p* = 0.052). The number of patients who developed grade 2 late GU toxicity with and without DM was 4 (16.7%) and 13 (5.7%) patients, respectively. In multivariate analysis, significant predictor for grade 2 late GU toxicity was not detected. No significant predictor for GI toxicity was detected.

**Table 3:** Predictive significance of clinical factors for late grade 2 toxicities

	Univariate			Multivariate		
	OR	(95% CI)	*p*-value	OR	(95% CI)	p-value
Late grade 2 GU toxicity (*n* = 17)						
Age	1.00	(0.93–1.07)	0.986	1.01	(0.93–1.09)	0.863
Risk group	0.68	(0.30–1.56)	0.363	1.31	(0.27–6.45)	0.741
ADT duration	0.98	(0.93–1.02)	0.337	0.96	(0.88–1.05)	0.407
DM	3.32	(0.99–11.15)	0.052	2.94	(0.80–10.82)	0.105
Anticoagulants	0.31	(0.04–2.38)	0.258	0.26	(0.03–2.28)	0.227
BPH	1.72	(0.36–8.14)	0.496	NA	–	–
TURP	15.60	(2.05–118.58)	**0.008**	NA	–	–
Late grade 2 GI toxicity (*n* = 3)						
Age	1.07	(0.90–1.29)	0.438	1.09	(0.0.88–1.35)	0.422
Risk group	NA	–	–	NA	–	–
ADT duration	1.02	(0.97–1.08)	0.403	0.98	(0.82–1.18)	0.850
DM	4.93	(0.43–56.53)	0.199	6.27	(0.42–94.06)	0.184
Anticoagulants	2.63	(0.23–29.64)	0.435	1.15	(0.08–16.27)	0.920
BPH	NA	–	–	NA	–	–
TURP	NA	–	–	NA	–	–

*OR* Odds ratios, *CI* Confidence interval, *NA* not available, *GU* genitourinary, *GU* gastrointesitinal, *ADT* androgen deprivation therapy, *DM* diabetes mellitus, *BPH* benign prostate hyperplasia, *TURP* transurethral resection of the prostate

## Discussion

We investigated the preliminary results of CIRT using the spot scanning method for prostate cancer in the present study. To the best of our knowledge, this is the first report about the clinical outcomes of prostate cancer patients after undergoing CIRT with the spot scanning method.

Late GI toxicity is often a problem with RT for prostate cancer. Technological improvements in RT, such as IMRT and particle therapy, can provide a better dose distribution to the target and spare the normal surrounding tissues. In patients with prostate cancer treated with high-dose 3DCRT, grade 2 or greater late GI toxicity was observed 14–24% of patients in a prior study [27–30]. Meanwhile, the rate of grade 2 or greater late GI toxicity was reduced to 5–15% using IMRT to spare the rectal dose [31–33].

Moreover, particle therapy can more strongly reduce the rectal dose than IMRT based on its sharp dose distribution to the target. According to results of a phase II clinical trial analyzing 84 patients treated with proton beam RT, the incidence of grade 2 late GI toxicity was 13% [34]. Iwata et al. reported the results of a multi-institutional retrospective survey of proton therapy for prostate cancer in Japan, and the incidence rate of grade 2 or greater severe late GI toxicity was 4.6% [35].

Late GI toxicity is also known as a dose-limiting factor in CIRT for prostate cancer. In a dose escalation study of CIRT for prostate cancer, grade 3 late GI toxicity developed in 36% of patients who received a dose of 72 Gy [8]. However, according to a phase II clinical study of CIRT for prostate cancer using a total dose of 66 Gy delivered in 20 fractions, grade 2 GI toxicity was observed in 2% of the patients [25]. Additionally, in a multi-institutional study of CIRT for prostate cancer, the incidence of grade 2 rectal toxicity was only 0.8% [13]. Similar results were obtained in the present study. In the study of the correlation between late GI toxicity and CIRT, anticoagulation therapy was associated with a 2.7-fold risk of late GI toxicity [36]. In the present study, significant correlation was not observed between anticoagulation therapy and late GI toxicity.

In this study, previous TURP was significantly associated with grade 2 late GU toxicity. A study of IMRT demonstrated that previous TURP was associated with late GU toxicity [37]. Another study of IMRT, DM was reported as a predictive factor for late grade 2 or greater GU toxicities [38]. In the present study, DM was tended to correlate with grade 2 late GU toxicity. In terms of late GU toxicity after CIRT, it was reported that longer ADT duration was a predictor of late GU toxicity [39]. However, in the present study, a significant correlation between ADT duration and late GU toxicity was not observed. Few studies have assessed the correlation between ADT duration and late GU toxicity, therefore, further studies are required to assess the relationship between CIRT and GU toxicity.

Several studies have demonstrated dose response in prostate cancer [4–7]. Only ADT is not sufficient for the definitive treatment for prostate cancer; high-dose radiation therapy is required [40]. On the basis of the very low α/β ratio for prostate cancer, hypofractionated radiotherapy would offer increased therapeutic benefit without increasing toxicity [41]. In fact, several studies have reported the efficacy of moderate hypofractionated RT for patients with prostate cancer [42–45]. Moreover, the clinical outcomes of extreme hypofractionated RT for those with prostate cancer have been recently reported [46–48]. According to these features of prostate cancer and because of the biological and physical advantages in CIRT, it is considered that CIRT is appropriate for the management of prostate cancer. In fact, favorable BFS rates have been reported in patients treated with CIRT. Ishikawa et al. reported a 5 year BFS rate of 90.6% for patients with prostate cancer treated with CIRT at a total dose of 66 Gy (RBE) delivered in 20 fractions [12]. In a multi-institutional analysis of CIRT, the 5 year BFS rates in the low-, intermediate-, and high-risk groups were 92, 89, and 92%, respectively [13]. In the present study, the BFS rate was worst in the low-risk group and best in the high-risk group. The two major reasons to explain these results are as follows. First, the number of the low-risk patients was small, i.e., only eight patients. Therefore, the BFS rate in the low-risk group seemed to be relatively higher than that in the other group; furthermore, there was only one case of biochemical relapse. One of these eight low-risk patients experienced PSA elevation immediately after CIRT, which may have been a benign temporary PSA elevation called PSA bounce; however, its significance was unclear owing to immediate ADT after PSA elevation without any radiological confirmation of clinical recurrence. Second, high-risk patients received ADT for a longer duration. In the present study, the high-risk group underwent ADT for a total of 2 years; thus, high-risk patients received ADT at least 1 year after the completion of CIRT. Therefore, the observation period was not sufficient to estimate the BFS rate in our study, and further observation will be necessary to confirm our treatment outcome.

In the present study, biochemical failure was observed in 14 patients, with PSA levels decreasing without treatment in 11 patients. PSA fluctuations without any clinical signs of cancer recurrence after RT follow-up are known as PSA bounces, and they are often observed after brachytherapy and/or external beam RT [49]. PSA bounces after low-dose brachytherapy occurs in 28–49% of patients using a 0.2 ng/ml definition [49–51]. In approximately 10% of patients, the PSA bounce exceeds the 2 ng/ml limit [51]. Age was one of the first and most frequently described predictive factors of the PSA bounce [49, 50]. A similar tendency was observed in the present study. There has been no study of PSA fluctuations after CIRT. The clinical significance of PSA fluctuations is unclear, and further study is required.

The present study had several limitations such as its single-institutional nature and short observation period. More than 80% of late toxicities occurred within 2 years after CIRT [37]; therefore, toxicities were evaluated for a sufficient period in the present study. Further observation with a large patient cohort will be necessary to confirm our clinical outcome.

## Conclusions

The present study demonstrated that CIRT using the spot scanning method for patients with prostate cancer has a favorable outcome. Further observation with a large patient cohort will be necessary to confirm our clinical outcome.

## Data Availability

The datasets used and/or analyzed during the current study are available from the corresponding author on reasonable request.

## Abbreviations

RT: Radiotherapy
IMRT: Intensity-modulated radiotherapy
CIRT: Carbon-ion radiotherapy
RBE: Relative biological effectiveness
i-ROCK: Ion beam Radiation Oncology Center in Kanagawa
KCC: Kanagawa Cancer Center
ADT: Androgen deprivation therapy
CT: Computed tomography
CTV: Clinical target volume
PTV: Planning target volume
PSA: Prostate specific antigen
BFS: Biochemical relapse-free survival
TURP: Transurethral resection of the prostate
GU: Genitourinary
GI: Gastrointestinal
DM: Diabetes mellitus

## References

[1] Bray F, Ferlay J, Soerjomataram Siegel RL, Torre LA, Jemal A (2018). Global cancer statistics 2018: GLOBOCAN estimates of incidence and mortality worldwide for 36 cancers in 185 countries. CA Cancer J Clin.

[2] Scherr D, Swindle PW, Scardino PT (2003). National Comprehensive Cancer Network guidelines for the management of prostate cancer. Urology..

[3] Hernandez DJ, Nielsen ME, Han M, Partin AW (2007). Contemporary evaluation of the D’amico risk classification of prostate cancer. Urology..

[4] Sanguineti G, Cavey ML, Endres EJ, Brandon GG, Bayouth JE (2006). Is IMRT needed to spare the rectum when pelvic lymph nodes are part of the initial treatment volume for prostate cancer?. Int J Radiat Oncol Biol Phys.

[5] Lyons JA, Kupelian PA, Mohan DS, Reddy CA, Klein EA (2000). Importance of high radiation doses (72 Gy or greater) in the treatment of stage T1–T3 adenocarcinoma of the prostate. Urology..

[6] Kupelian PA, Mohan DS, Lyons J, Klein EA, Reddy CA (2000). Higher than standard radiation doses (72 Gy) with or without androgen deprivation in the treatment of localized prostate cancer. Int J Radiat Oncol Biol Phys.

[7] Kupelian PA, Buchsbaum JC, Reddy CA, Klein EA (2001). Radiation dose response in patients with favorable localized prostate cancer (stage T1–T2, biopsy Gleason < or = 6, and pretreatment prostate-specific antigen < or =10). Int J Radiat Oncol Biol Phys.

[8] Akakura K, Tsujii H, Morita S, Tsuji H, Yagishita T, Isaka S, Ito H, Akaza H, Hata M, Fujime M, Harada M, Shimazaki J (2004). Phase I/II clinical trials of carbon ion therapy for prostate cancer. Prostate.

[9] Kanai T, Endo M, Minohara S, Miyahara N, Koyama-ito H, Tomura H, Matsufuji N, Futami Y, Fukumura A, Hiraoka T, Furusawa Y, Ando K, Suzuki M, Soga F, Kawachi K (1999). Biophysical characteristics of HIMAC clinical irradiation system for heavy-ion radiation therapy. Int J Radiat Oncol Biol Phys.

[10] Kanai T, Matsufuji N, Miyamoto T, Mizoe J, Kamada T, Tsuji H, Kato H, Baba M, Tsujii H (2006). Examination of GyE system for HIMAC carbon therapy. Int J Radiat Oncol Biol Phys.

[11] Schulz-Ertner D, Tsujii H (2007). Particle radiation therapy using proton and heavier ion beams. J Clin Oncol.

[12] Ishikawa H, Tsuji H, Kamada T, Akakura K, Suzuki H, Shimazaki J, Tsujii H (2012). Carbon-ion radiation therapy for prostate cancer. Int J Urol.

[13] Nomiya T, Tsuji H, Kawamura H, Ohno T, Toyama S, Shioyama Y, Nakayama Y, Nemoto K, Tsujii H, Kamada T (2016). A multi-institutional analysis of prospective studies of carbon-ion radiotherapy for prostate cancer: a report from the Japan carbon-ion radiation oncology study group (J-CROS). Radiother Oncol.

[14] Nakayama Y, Minohara S, Nonaka T, Nomiya T, Kusano Y, Takeshita E, Mizoguchi N, Hagiwara Y (2016). The ion-beam radiation oncology Center in Kanagawa (i-ROCK) carbon ion Facility at the Kanagawa Cancer Center. Int J Part Ther.

[15] Tsujii H, Kamada T, Shirai T, Noda K, Tsuji H, Karasawa K (2014). Carbon-ion radiotherapy.

[16] Minohara S, Fukuda S, Kanematsu N, Takei Y, Furukawa T, Inaniwa T, Matsufuji N, Mori S, Noda K (2010). Recent innovations in carbon-ion radiotherapy. J Radiat Res.

[17] Ute L (2012). Ion Beam Therapy.

[18] Weber U, Becher W, Kraft G (2000). Depth scanning for a conformal ion beam treatment of deep seated tumours. Phys Med Biol.

[19] Pedroni E, Bacher R, Blattmann H, Böhringer T, Coray A, Lomax A, Lin S, Munkel G, Scheib S, Schneider U (1995). The 200-MeV proton therapy project at the Paul Scherrer Institute: conceptual design and practical realization. Med Phys.

[20] Chu WT, Ludewigt BA, Renner TR (1993). Instrumentation for treatment of cancer using proton and light-ion beams. Rev Sci Instrum.

[21] D'Amico AV, Whittington R, Malkowicz SB, Schultz D, Blank K, Broderick GA, Tomaszewski JE, Renshaw AA, Kaplan I, Beard CJ, Wein A (1998). Biochemical outcome after radical prostatectomy, external beam radiation therapy, or interstitial radiation therapy for clinically localized prostate cancer. JAMA..

[22] Kawamura H, Kubo N, Sato H, Mizukami T, Katoh H, Ishikawa H, Ohno T, Matsui H, Ito K, Suzuki K, Nakano T (2020). Group for Genitourinary Tumors at Gunma University Heavy Ion Medical Center. Moderately hypofractionated carbon ion radiotherapy for prostate cancer; a prospective observational study “GUNMA0702”. BMC Cancer.

[23] Kasuya G, Ishikawa H, Tsuji H, Haruyama Y, Kobashi G, Ebner DK, Akakura K, Suzuki H, Ichikawa T, Shimazaki J, Makishima H, Nomiya T, Kamada T (2017). Tsujii H; working Group for Genitourinary Tumors. Cancer-specific mortality of high-risk prostate cancer after carbon-ion radiotherapy plus long-term androgen deprivation therapy. Cancer Sci.

[24] Tsuji H, Yanagi T, Ishikawa H, Kamada T, Mizoe JE, Kanai T, Morita S, Tsujii H (2005). Hypofractionated radiotherapy with carbon ion beams for prostate cancer. Int J Radiat Oncol Biol Phys.

[25] Ishikawa H, Tsuji H, Kamada T, Yanagi T, Mizoe JE, Kanai T, Morita S, Wakatsuki M, Shimazaki J, Tsujii H (2006). Carbon ion radiation therapy for prostate cancer: results of a prospective phase II study. Radiother Oncol.

[26] Roach M, Hanks G, Thames H, Schellhammer P, Shipley WU, Sokol GH, Sandler H (2006). Defining biochemical failure following radio therapy with clinically localized prostate cancer:recommendations of the RTOG- ASTRO Phoenix consensus conference. Int J Radiation Oncol Biol Phys.

[27] Hanks GE (2000). Conformal radiotherapy for prostate cancer. Ann Med.

[28] Vargas C, Martinez A, Kestin LL, Yan D, Grills I, Brabbins DS, Lockman DM, Liang J, Gustafson GS, Chen PY, Vicini FA, Wong JW (2005). Dose-volume analysis of predictors for chronic rectal toxicity after treatment of prostate cancer with adaptive image-guided radiotherapy. Int J Radiat Oncol Biol Phys.

[29] Pollack A, Zagars GK, Starkschall G, Antolak JA, Lee JJ, Huang E, von Eschenbach AC, Kuban DA, Rosen I (2002). Prostate cancer radiation dose response: results of the M. D. Anderson phase III randomized trial. Int J Radiat Oncol Biol Phys.

[30] Zelefsky MJ, Cowen D, Fuks Z, Shike M, Burman C, Jackson A, Venkatramen ES, Leibel SA (1999). Long term tolerance of high dose three-dimensional conformal radiotherapy in patients with localized prostate carcinoma. Cancer.

[31] Sveistrup J, af Rosenschöld PM, Deasy JO, Oh JH, Pommer T, Petersen PM, Engelholm SA (2014). Improvement in toxicity in high risk prostate cancer patients treated with image-guided intensity-modulated radiotherapy compared to 3D conformal radiotherapy without daily image guidance. Radiat Oncol.

[32] Michalski JM, Yan Y, Watkins-Bruner D, Bosch WR, Winter K, Galvin JM, Bahary JP, Morton GC, Parliament MB, Sandler HM (2013). Preliminary toxicity analysis of 3-dimensional conformal radiation therapy versus intensity modulated radiation therapy on the high-dose arm of the radiation therapy oncology group 0126 prostate cancer trial. Int J Radiat Oncol Biol Phys.

[33] Kupelian PA, Willoughby TR, Reddy CA, Klein EA, Mahadevan A (2007). Hypofractionated intensity-modulated radiotherapy (70 Gy at 2.5 Gy per fraction) for localized prostate cancer: Cleveland Clinic experience. Int J Radiat Oncol Biol Phys.

[34] Coen JJ, Bae K, Zietman AL, Patel B, Shipley WU, Slater JD, Rossi CJ (2011). Acute and late toxicity after dose escalation to 82 GyE using conformal proton radiation for localized prostate cancer: initial report of American College of Radiology Phase II study 03–12. Int J Radiat Oncol Biol Phys.

[35] Iwata H, Ishikawa H, Takagi M, Okimoto T, Murayama S, Akimoto T, Wada H, Arimura T, Sato Y, Araya M, Mizoe JE, Gosho M, Nakamura K, Shirato H, Sakurai H (2018). Long-term outcomes of proton therapy for prostate cancer in Japan: a multi-institutional survey of the Japanese radiation oncology study group. Cancer Med.

[36] Ishikawa Hitoshi, Tsuji Hiroshi, Kamada Tadashi, Hirasawa Naoki, Yanagi Takeshi, Mizoe Jun-Etsu, Akakura Koichiro, Suzuki Hiroyoshi, Shimazaki Jun, Tsujii Hirohiko (2006). Risk factors of late rectal bleeding after carbon ion therapy for prostate cancer. International Journal of Radiation Oncology*Biology*Physics.

[37] Byrne K, Hruby G, Kneebone A, Whalley D, Guo L, McCloud P, Eade T (2017). Late genitourinary toxicity outcomes in 300 prostate cancer patients treated with dose-escalated image-guided intensity-modulated radiotherapy. Clin Oncol (R Coll Radiol).

[38] Kalakota K, Liauw SL (2013). Toxicity after external beam radiotherapy for prostate cancer: an analysis of late morbidity in men with diabetes mellitus. Urology..

[39] Ishikawa H, Tsuji H, Kamada T, Hirasawa N, Yanagi T, Mizoe JE, Akakura K, Suzuki H, Shimazaki J, Nakano T, Tsujii H (2008). Adverse effects of androgen deprivation therapy on persistent genitourinary complications after carbon-ion radiotherapy for prostate cancer. Int J Radiat Oncol Biol Phys.

[40] Nomiya T, Tsuji H, Toyama S, Maruyama K, Nemoto K, Tsujii H, Kamada T (2013). Management of high-risk prostate cancer: radiation therapy and hormonal therapy. Cancer Treat Rev.

[41] Hausterman K, Fowler JF (2000). A comment on proliferation rates in human prostate cancer. Int J Radiat Oncol Biol Phys.

[42] Aluwini S, Pos F, Schimmel E, Krol S, van der Toorn PP, de Jager H, Alemayehu WG, Heemsbergen W, Heijmen B, Incrocci L (2016). Hypofractionated versus conventionally fractionated radiotherapy for patients with prostate cancer (HYPRO): late toxicity results from a randomised, non-inferiority, phase 3 trial. Lancet Oncol.

[43] Dearnaley D, Syndikus I, Mossop H, Khoo V, Birtle A, Bloomfield D, Graham J, Kirkbride P, Logue J, Malik Z, Money-Kyrle J, O'Sullivan JM, Panades M, Parker C, Patterson H, Scrase C, Staffurth J, Stockdale A, Tremlett J, Bidmead M, Mayles H, Naismith O, South C, Gao A, Cruickshank C, Hassan S, Pugh J, Griffin C, Hall E, CHHiP Investigators (2016). Conventional versus hypofractionated high-dose intensity-modulated radiotherapy for prostate cancer: 5-year outcomes of the randomised, non-inferiority, phase 3 CHHiP trial. Lancet Oncol.

[44] Catton CN, Lukka H, Gu CS, Martin JM, Supiot S, Chung PWM, Bauman GS, Bahary JP, Ahmed S, Cheung P, Tai KH, Wu JS, Parliament MB, Tsakiridis T, Corbett TB, Tang C, Dayes IS, Warde P, Craig TK, Julian JA, Levine MN (2017). Randomized trial of a Hypofractionated radiation regimen for the treatment of localized prostate Cancer. J Clin Oncol.

[45] Takakusagi Y, Kawamura H, Okamoto M, Kaminuma T, Kubo N, Mizukami T, Sato H, Onishi M, Ohtake N, Sekihara T, Nakano T (2019). Long-term outcome of hypofractionated intensity-modulated radiotherapy using TomoTherapy for localized prostate cancer: a retrospective study. PLoS One.

[46] Zilli T, Franzese C, Bottero M, Giaj-Levra N, Förster R, Zwahlen D, Koutsouvelis N, Bertaut A, Blanc J, Roberto D'agostino G, Alongi F, Guckenberger M, Scorsetti M, Miralbell R (2019). Single fraction urethra-sparing prostate cancer SBRT: phase I results of the ONE SHOT trial. Radiother Oncol.

[47] Alongi F, Fiorentino A, De Bari B (2015). SBRT and extreme hypofractionation: a new era in prostate cancer treatments?. Rep Pract Oncol Radiother.

[48] Nicosia L, Mazzola R, Rigo M, Figlia V, Giaj-Levra N, Napoli G, Ricchetti F, Corradini S, Ruggieri R, Alongi F (2019). Moderate versus extreme hypofractionated radiotherapy: a toxicity comparative analysis in low- and favorable intermediate-risk prostate cancer patients. J Cancer Res Clin Oncol.

[49] Burchardt W, Skowronek J (2018). Time to PSA rise differentiates the PSA bounce after HDR and LDR brachytherapy of prostate cancer. J Contemp Brachytherapy.

[50] Crook J, Gillan C, Yeung I, Austen L, McLean M, Lockwood G (2007). PSA kinetics and PSA bounce following permanent seed prostate brachytherapy. Int J Radiat Oncol Biol Phys.

[51] Caloglu M, Ciezki J (2009). Prostate-specific antigen bounce after prostate brachytherapy: review of a confusing phenomenon. Urology.

