# Correction to: Preliminary result of carbon-ion radiotherapy using the spot scanning method for prostate cancer

**DOI:** 10.1186/s13014-022-02032-3

**Published:** 2022-05-23

**Authors:** Yosuke Takakusagi, Hiroyuki Katoh, Kio Kano, Wataru Anno, Keisuke Tsuchida, Nobutaka Mizoguchi, Itsuko Serizawa, Daisaku Yoshida, Tadashi Kamada

**Affiliations:** Department of Radiation Oncology, Kanagawa Cancer Center, Asahi-ku, Yokohama, Kanagawa 241-8515 Japan

## Correction to: Radiation Oncology (2020) 15:127 https://doi.org/10.1186/s13014-020-01575-7

Following publication of the original article [1], the authors identified minor errors that should be addressed.

In the **Abstract**, the original publication read (affected area marked in bold):

Grade 2 acute rectal toxicity was not observed. Grade 2 late urinary toxicity and grade 2 late rectal toxicity were observed in **17 (6.7%)** and 3 patients (1.2%), respectively.

The corrected sentence reads:

Grade 2 acute rectal toxicity was not observed. Grade 2 late urinary toxicity and grade 2 late rectal toxicity were observed in **16 (6.3%)** and 3 patients (1.2%), respectively.

In **Table 1**, Row 1, the Follow-up duration, months, median (range) originally read 24.3 (4.1–39.5). The corrected reading is 35.3 (4.1–52.9).

The corrected Table 1 is given here.

**Table 1:** Patient characteristics (*n* = 253)

Characteristics	n (%)
Follow-up duration, months, median (range)	35.3 (4.1–52.9)
Age, years, median (range)	70 (47–86)
T stage	
1c	49 (19.4%)
2a	79 (31.2%)
2b	35 (13.8%)
2c	53 (20.9%)
3a	27 (10.7%)
3b	10 (4.0%)
Pretreatment PSA, ng/ml, median (range)	8.6 (3.33–187)
< 10	147 (58.1%)
10 ≤ 20	73 (28.9%)
20 ≤	33 (13.0%)
Gleason score	
6	14 (5.5%)
7	117 (46.2%)
8	79 (31.2%)
9	43 (17.0%)
10	0 (0.0%)
D’Amico classification	
low	8 (3.2%)
intermediate	88 (34.8%)
high	157 (62.1%)
ADT	
none	9 (3.6%)
neoadjuvant	87 (34.4%)
neoadjuvant and adjuvant	157 (62.1%)
Complications, histories	
Diabetes mellitus	25 (9.9%)
Internal use of anticoagulants	41 (16.2%)
Benign prostate hyperplasia	18 (7.1%)
TURP	4 (1.6%)

*PSA* prostate specific antigen, *ADT* androgen deprivation therapy, *TURP* transurethral resection of the prostate

In **Results, Toxicities,** the original publication read (affected area marked in bold):

Grades 1 and 2 urinary frequency were observed in **36 (14.8%) and 12 (4.7%)** patients, respectively.

The corrected sentence reads:

Grades 1 and 2 urinary frequency were observed in **34 (13.4%) and 9 (3.6%)** patients, respectively.

In **Results, Toxicities,** the original publication read (affected areas marked in bold):

The late GU toxicity grades were one in 52 patients (20.6%) and **two in 17 patients (6.7%)**. Grade 3 or greater late GU toxicity was not observed. Among the late GU toxicities, grades 1 and 2 hematuria were observed in 14 (5.5%) and one patient (0.4%), respectively. Grades 1 and 2 urinary frequency were observed in **28 (11.1%)** and 11 (4.3%) patients, respectively, and grades 1 and 2 urinary stricture were observed in 5 (2.0%) and 3 (1.2%) patients, respectively.

The corrected sentences read:

The late GU toxicity grades were one in 52 patients (20.6%) and **two in 16 patients (6.3%)**. Grade 3 or greater late GU toxicity was not observed. Among the late GU toxicities, grades 1 and 2 hematuria were observed in 14 (5.5%) and one patient (0.4%), respectively. Grades 1 and 2 urinary frequency were observed in **26 (10.3%)** and 11 (4.3%) patients, respectively, and grades 1 and 2 urinary stricture were observed in 5 (2.0%) and 3 (1.2%) patients, respectively.

In the **Discussion**, an incorrect reference number was used; [37] has been replaced by the correct [36] in the following sentence:

"More than 80% of late toxicities occurred within 2 years after CIRT [36];..."

The correction does not have any effect on the final conclusions of the paper. The original article has been corrected.
